# Expression of antigen tf and galectin-3 in fibroadenoma

**DOI:** 10.1186/1756-0500-5-694

**Published:** 2012-12-24

**Authors:** Itandehui Belem Gallegos, Eduardo Pérez-Campos, Margarito Martinez, Miguel Ángel Mayoral, Laura Pérez, Sergio Aguilar, Edgar Zenteno, Maria del Socorro Pina, Pedro Hernández

**Affiliations:** 1 Centro de Investigaciones en Ciencias Medicas y Biológicas Facultad de Medicina, Universidad Autónoma Benito Juárez de Oaxaca, 68020, Oaxaca, Mexico; 2 Unidad de Investigación en Bioquímica, Instituto Tecnológico de Oaxaca, Oaxaca, Mexico; 3 Laboratorio de Inmunología, Departamento de Bioquímica, Facultad de Medicina, Universidad Nacional Autónoma de México, Mexico, 04510, Mexico

**Keywords:** Antigen TF, Galectin-3, Fibroadenoma, Breast cancer, Plant lectins

## Abstract

**Background:**

Fibroadenomas are benign human breast tumors, characterized by proliferation of epithelial and stromal components of the terminal ductal unit. They may grow, regress or remain unchanged, as the hormonal environment of the patient changes. Expression of antigen TF in mucin or mucin-type glycoproteins and of galectin-3 seems to contribute to proliferation and transformations events; their expression has been reported in ductal breast cancer and in aggressive tumors.

**Findings:**

Lectin histochemistry, immunohistochemistry, and immunofluorescence were used to examine the expression and distribution of antigen TF and galectin-3. We used lectins from *Arachis hypogaea, Artocarpus integrifolia,* and *Amaranthus lecuocarpus* to evaluate TF expression and a monoclonal antibody to evaluate galectin-3 expression. We used paraffin-embedded blocks from 10 breast tissues diagnosed with fibroadenoma and as control 10 healthy tissue samples. Histochemical and immunofluorescence analysis showed positive expression of galectin-3 in fibroadenoma tissue, mainly in stroma, weak interaction in ducts was observed; whereas, in healthy tissue samples the staining was also weak in ducts. Lectins from A. *leucocarpus* and *A. integrifolia* specificaly recognized ducts in healthy breast samples, whereas the lectin from *A. hypogaea* recognized ducts and stroma. In fibroadenoma tissue, the lectins from *A. integrifolia*, *A. Hypogaea,* and *A. leucocarpus* recognized mainly ducts.

**Conclusions:**

Our results suggest that expression of antigen TF and galectin-3 seems to participate in fibroadenoma development.

## Background

Fibroadenomas are benign breast tumors commonly found in young women. Fibroadenoma is a biphasic lesion of the breast characterized by proliferation of both epithelial and stromal components of the terminal ductal unit. Proliferation of stromal cells is commonly considered the primary event in the development of a fibroadenoma, followed by secondary proliferation of epithelial cells [1]. Most fibroadenomas are considered to be the result of hyperplastic processes involving connective tissue of lobular units [2]. Fibroadenomas’ development is heterogeneous, since they may grow, regress, or remain unchanged as the hormonal environment ofthe patient changes, but most stop growing after reaching 2 to 3 cm in diameter [3], moreover, with aging, the stroma becomes less cellular and increases its hyalinization [4]. Occurrence in young women and sclerotic involution in the elderly suggest a hormonal responsiveness of fibroadenomas [1].

O-glycosylation plays an important role in the biological activity of glycoproteins involved in controlling cell differentiation [5,6]. Alterations in glycosylation of cell membrane glycocongugates in neoplastic lesions from a variety of organs, including lung, stomach, ovary, skin and endometrium, have been reported [7,8]. Abnormal O-glycosylation, especially in mucin and mucin type glyproteins, results in exposure of the peptide core, as well as in the exposure of the normally cryptic core TF (Galβ1-3GalNAcα1-O-Ser/Thr) antigen [9], which is distributed discontinuously along the peptide backbone, and premature sialylation can occur leading to formation of antigens related to cancer progression [10].

Lectins are proteins that recognize carbohydrates or precipitate glycoconjugates and they are important tools for oligosaccharide characterization as well as for isolation of cellular populations [11]. Galectin-3 is a 31 kDa protein member of the beta-galactoside-binding proteins; it is an intracellular and extracellular lectin that interacts with intracellular glycoproteins, cell surface molecules, and extracellular matrix proteins. Galectin-3 is expressed widely in epithelial and immune cells and its expression is correlated with cancer aggressiveness and metastasis [12]. The aim of this study was to determine, by histochemsitry, the over-expression of antigen TF and galectin-3 in fibroadenoma and healthy breast tissues, using specific lectins for antigen TF and anti-galectin-3 antibody, to understand better the potential role of O-glycosylation in fibroadenomas’ progression.

## Findings

### Reagents

Biotynilated lectins from *Arachis hypogaea* and *Artocarpus integrifolia were* obtained from Vector Laboratories (Burlingame, CA USA). Lectin from *Amaranthus leucocarpus* (ALL) was purified by affinity chromatography using a column containing stroma from human O-desialylated erythrocytes entrapped in Sephadex G-25 (Upssula Sweden), as described previously [13]. ALL was labeled with the N-hydroxisuccinimide ester of biotin (Bio-Rad Inc., Richmond, CA, USA) at a label/protein ratio of 2:1 [14] Avidin-peroxidase, sugars, and chemical reagents were from (Sigma Chemical Co, St. Louis, MO, USA), 3-amino-9-ethyl-carbazole (AEC) kit used as substrate for peroxidase was obtained from Vector Laboratories. Biotin-labeled mouse anti-galectin-3 was obtained from Invitrogen (Carlsbad, CA USA).

### Source of tissues

Ten paraffin-embedded blocks from breast tissues diagnosed with fibroadenoma were kindly donated by Paulina Leyva, from the Pathology Department of the School of Medicine, UABJO, Oaxaca, Mexico. Ten healthy control tissue samples were obtained from cosmetic procedures at the Plastic Surgery service from the Mexican Institute of Social Security (IMSS, for its initials in Spanish), Mexico.

### Ethical approval

The study protocol was approved by the Institutional Review Board of Research of the Medical School of UABJO.

### Immunohistochemistry

Paraffin-embedded blocks from fibroadenoma and normal breast tissues, the latter used as controls, were cut in 6-μm-thick sections. Sections were incubated with each biotin-labeled lectin (1 μg/ml) or monoclonal anti-galectin-3 antibody (dilution 1:100), overnight at 4°C. After incubation, the slides were washed with PBS, pH 7.4, and covered with 300 μl of 5% skimmed milk in PBS, pH 7.4, and incubated for 12 h at 4°C. Then, after washing with PBS, pH 7.4, the samples were labeled with streptavidin-peroxidase (1:1000 in PBS) for 1 h at 37°C. Unbound conjugate was removed by washing six times with PBS. The binding of lectins or antibody was revealed by incubating with 3-amino-9-ethyl-carbazole (AEC), following instructions of manufacturer (Invitrogen), during 15 min at 37°C. The reaction was stopped by washing with water. Slides were observed with an AXIOSCOP 40 microscope (Zeiss, Germany) equipped with a digital camera AXIOCAM MRC (Zeiss) and micrographs were analyzed with the AXIOVISION 4.3 Software (Zeiss).

### Immunofluorescence

Double labeling of slides was performed as follows: Tissue samples were labeled with lectins (1 μg/ml) overnight at 4°C and monoclonal anti-galectin-3 antibody used at 1:100 following the same procedure as previously described, except that lectin binding was indirectly recognized with extravidin-FITC conjugated (Sigma Chemical Co.) and visualized using a green filter. Anti-galectin antibodies were revealed with extravidin-red-X conjugate (Invitrogen) and visualized using a red filter. Slides were observed with an AXIOSCOP 40 microscope (Zeiss), equipped with a digital camera AXIOCAM MRC (Zeiss) and micrographs were analyzed with the AXIOVISION 4.3 Software (Zeiss).

### Lectin specificity

To determinate the lectins’ specificity in control breast epithelium and fibroadenomas, lectin histochemistry and immunoflourescence assays were performed using lectins incubated with 200 mM of their specific monosaccharide (N-acetyl-D-galactosamine) 30 min before use.

### Statistical analysis

Fisher’s exact test using Woolf’s approximation was performed using GraphPad InStat version 3.00, GraphPad Software, San Diego California USA.

### Results

#### Lectins and anti galectin-3 histochemistry

**Figure 1:**
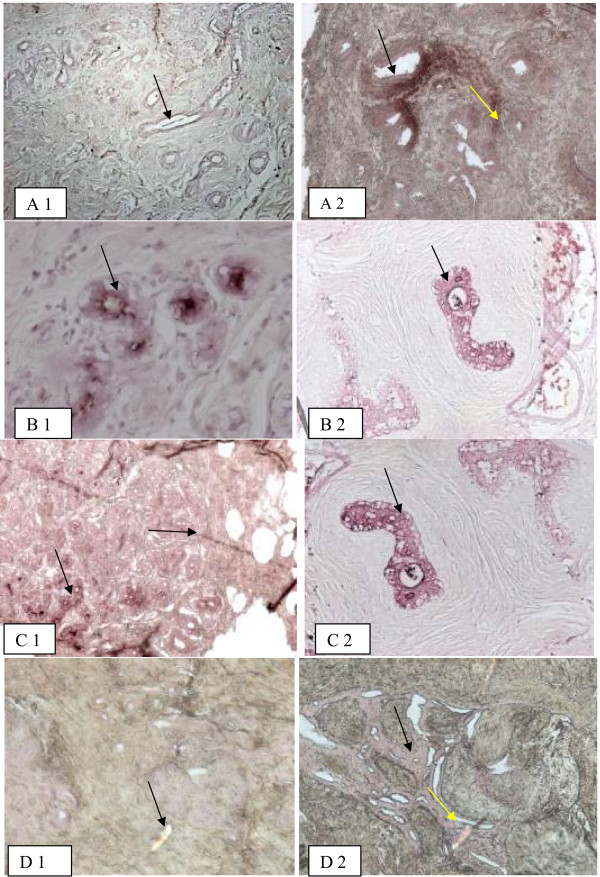
**Lectin and anti-galectin-3 histochemistry in healthy breast and fibroadenoma samples. ***Amaranthus leucocarpus* recognizes ducts in healthy breast samples (**A**1): in fibroadenoma, *Amaranthus leucocarpus* lectin recognizes ducts and stroma cells (**A**2)*. Artocarpus integrifolia* lectin recognizes ducts in healthy (**B**1) and fibroadenoma samples (**B**2)*. Arachis hypogaea* recognizes ducts in healthy (**C**1) and in fibroadenoma samples (**C**2). Anti-galectin-3 antibody depicts weak staining in ducts of healthy samples (**D**1); in fibroadenoma, the antibody recognizes ducts and stroma cells (**D**2) Arrows indicate the lectin and antibody binding sites. Black arrow indicates ducts site of lectin or antibody binding. Yellow arrow indicates stroma site of lectin or antibody binding. Micrographs are in 10X.

Numbers of samples positive and negative to either lectins or antibody are summarized in Table 1. As indicated in Table 2, in control samples, obtained from healthy tissues. *Amaranthus leucocarpus* lectin (ALL) recognized ducts in healthy breast samples (Figure 1. A1); whereas, in fibroadenoma tissues, this lectin recognized ducts and stroma cells (Figure 1. A2)*. A. integrifolia* lectin recognized ducts in healthy (Figure 1. B1) and fibroadenoma samples equally well (Figure 1. B2)*. A. hypogaea* recognized ducts in healthy (Figure 1. C1) and fibroadenoma samples (Figure 1. C2). Anti-galectin-3 antibody showed a weak staining in ducts of healthy samples (Figure 1. D1); however, in fibroadenoma tissue, the antibody recognized ducts and stroma cells (Figure 1. D2).

**Table 1:** Interaction of lectin or antibodies with all the healthy and fibroadenoma breast samples* used in this study

**ANTIBODY**	**NORMAL**		**FIBROADENOMA**	
	**+**	**-**	**+**	**-**
Anti galectin-3	10	0	10	0
*Arachis hypogaea*	10	0	10	0
*Artocarpus integrifolia*	6	4	8	2
*Amaranthus leucocarpus*	6	4	8	2

N = 10 healthy and 10 fibroadenoma samples evaluated.

**Table 2:** Staining of normal and fibroadenoma using anti-galectin-3 antibody and lectins*

**ANTIBODY**	**NORMAL**	**FIBROADENOMA**
Anti-Galectin-3	1	2
*Arachis hypogaea*	2	1
*Artocarpus integrifolia*	1	1
*Amaranthus leucocarpus*	1	2

*The presence and absence of staining in breast samples was recorded as follows: No stain, 0; ductal stain, 1; ductal and estromal stain, 2.

#### Immunofluorescence

**Figure 2:**
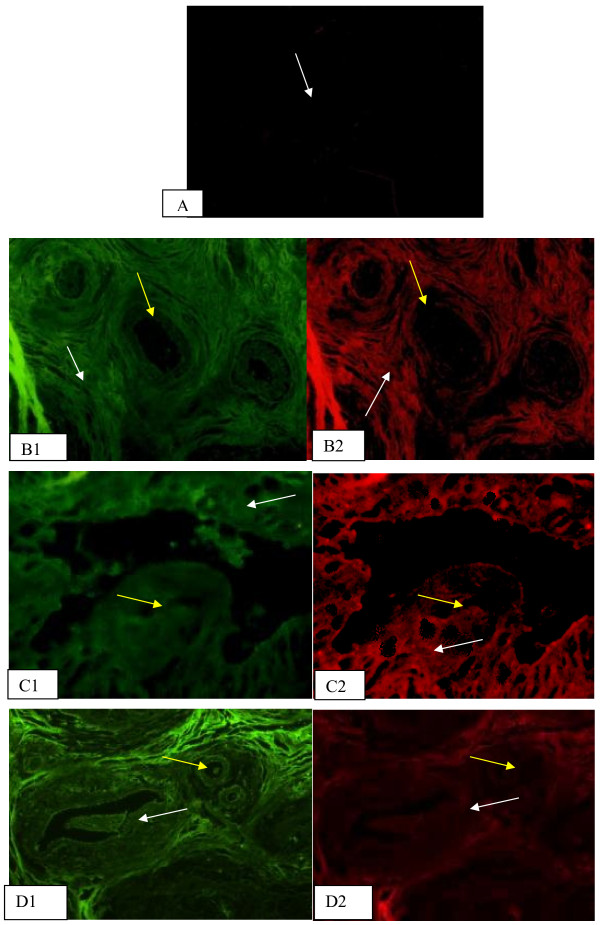
**Lectin and anti-galectin-3 double labeling histochemistry, in healthy breast and fibroadenoma samples.** Weak staining with anti-galectin-3 was observed in healthy samples (**A**). *Artocarpus integrifolia* lectin recognizes ducts and stroma in healthy breast samples (**B**1). *Arachis hypogaea* recognizes ducts and stroma in healthy samples (**C**1). *Amaranthus leucocarpus* recognizes ducts and stroma in healthy breast samples (**D**1). In fibroadenoma, anti-galectin-3 recognizes ducts and stroma (**B**2) (**C**2) (**D**2). White arrow indicates ducts site of lectin or antibody binding. Yellow arrow indicates stroma site of lectin or antibody binding. Micrographs 10X.

**Figure 3:**
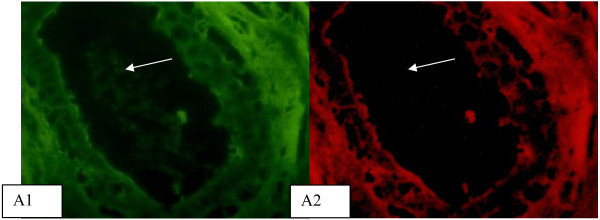
**Lectin and anti-galectin-3 double labeling histochemistry in fibroadenoma samples.***Artocarpus integrifolia* lectin recognizes luminal cells of ducts (**A**1). No interaction with luminal cells was observed in fibroadenoma using anti-galectin-3 (**A**2). White arrow indicates ducts site of lectin or antibody binding. Micrographs (40X).

Lectins and anti-galectin-3, in double labeling immunoflourescence, in healthy breast and fibroadenoma samples, showed weak staining with anti-galectin-3 in healthy samples (Figure 2. A). *A. integrifolia* lectin recognized ducts and stroma in healthy (Figure 2. B1) and in fibroadenoma samples; whereas antigalectin-3 recognized ducts and stroma (Figure 2. B2). *A. hypogaea* recognized ducts and stroma in healthy samples (Figure 2. C1); whereas, in fibroadenoma samples, anti-galectin-3 recognized ducts and stroma (Figure 2. C2). *Amaranthus leucocarpus* recognized ducts and stroma in healthy breast samples (Figure 2. D1); in fibroadenoma samples, anti-galectin-3 recognized ducts and stroma (Figure 2. D2). *A. integrifolia* lectin recognized luminal cells of ducts in fibroadenoma (Figure 3. A1). No interaction with luminal cells was observed in fibroadenomas using anti-galectin-3 antibody (Figure 3. A2). Lectins and anti-galectin-3 antibody staining showed no co-localization.

#### Statistical results

Lectins and using anti-galectin-3 antibody were not statistically significant

### Discussion

A fibroadenoma is a benign tumor with stromal and epithelial elements [15,16]; however, it has been associated with increased risk for breast cancer, particularly when associated with fibrocystic changes, proliferative breast disease, or a family history of breast cancer [17]. Recently, studies in alterations of the membrane’s protein glycosylation have been performed to understand better the changes taking place during cellular transformation to cancer [18,19]. Lectins, due to their higher specificity for carbohydrates and glycoconjugates, have been used to detect glycosylation changes in cancer cells [20-22]. In this work, we studied the glycosylation pattern in fibroadenomas using lectins with specificity for N-acetyl-D-galactosamine linked to protein or lipids. In fibroadenoma samples, lectins recognized different cytoplasmic regions from those recognized by antibodies, indicating that some cells express mucin-type O-glycans. In dermal carcinoma, as well as in carcinoma *in situ*[23], *Arachis hypogaea, Artocarpus integrifolia*, and *Amaranthus leucocarpus* lectins recognize the Galβ1-3GalNAc or TF antigen (Thomsen-Friedenreich antigen). Our results showed that the *A. leucocarpus* lectin recognized ducts in control samples; whereas, in fibroadenoma, it recognized ducts and some stromal cells. The recognition pattern of *Arachis hypogaea* was the same in control and fibroadenoma tissues, i.e., the lectin recognized ducts. *A. intergrifolia* recognized ducts in control samples, but in fibroadenoma the lectin recognized luminal cells. The ability of lectins to bind carbohydrates depends on their 3-D structure [24,25] and on their capacity to detect subtle variations in the conformation of carbohydrate structures of cell surfaces [25]. This ability could be explained by the variability in the size of the carbohydrate-recognition domain (CDR) and the variability in quaternary association [25]. Interestingly, the CDR of *A. leucocarpus* lectin recognizes GalNAc residues when they are spaced out in glycan structures, whereas GalNAc residues arranged in clusters prevent interaction with the lectin [26]. These glycans have been related with cervical cancer development [27] and are present in fibroadenomas [28], whereas *Artocarpus integrifolia* lectin can recognize clusters of TF antigen.

Galectin-3 is a naturally occurring galactoside-binding lectin expressed intra- and extra-cellularly by many cell types [29]. It has been shown that galectin-3 expression is increased in patients with breast, gastrointestinal, or lung cancer [30]. Moreover, higher galectin-3 expression has been shown in patients with metastatic disease than in patients with localized tumors [31]. Cytoplasmic galectin-3 is known to be anti-apoptotic, whereas nuclear galectin-3 promotes pre-mRNA splicing [32]. Cell surface galectin-3 is involved in various cell-cell and cell-matrix interactions [33,34] and enhances cancer cell adhesion and invasion through basement membrane by interacting with extracellular matrix proteins such as fibronectin, collagen, or laminin [35,36]. Galectin-3 expressed on the endothelial cell surface has been shown to promote adhesion of breast cancer cells to the endothelium by interaction with cancer- associated Thomsen-Friedenreich antigen cell surface molecules [37,38]. TF antigen is the core I structure of mucin-type *O*-linked glycans, but in its simplest nonsialylated form, as non-extended form it acts as an oncofetal antigen, and its presence/expression is increased in malignant and premalignant epithelia [39,40]. A weak interaction with ducts, in healthy samples was observed when anti-galectin-3 antibody was used, whereas, in fibroadenoma samples, the interaction was observed in ducts and stromal cells.

### Conclusions

Our results suggest that galectin-3 and Galß1,3-GalNAC glycosylated glycoproteins represent important elements in fibroadenomas’ development, reinforcing the notion that lectins constitute a very useful tool for the study of breast cancer.

## Abbreviations

AEC: 3-amino-9-ethyl-carbazole; CDR: Carbohydrate-recognition domain; FITC: Fluorescein isothiocyanate; Gal: Galactose; GalNAc: N-acetylgalactosamine; PBS: Phosphate buffered saline; Ser: Serine; Thr: Threonine; TF: Thomsen-Friedenreich antigen; UABJO: Universidad Autónoma “Benito Juárez” de Oaxaca; UNAM: Universidad Autónoma de México; USA: United Satates of America.

## Competing interests

The authors declare that they have no competing interest

## Authors’ contributions

IBG processed the samples, analyzed data, and reviewed the literature. EP analyzed data and reviewed the manuscript. PH performed literature review, drafted most of the manuscript. EZ analyzed data and reviewed the manuscript. SA, MM, MAM and LP reviewed the manuscript. All authors have read and approved the final manuscript.
